# *Plasmodium* immunotherapy combined with gemcitabine has a synergistic inhibitory effect on tumor growth and metastasis in murine Lewis lung cancer models

**DOI:** 10.3389/fonc.2023.1181176

**Published:** 2023-10-17

**Authors:** Xiao Chen, Zhu Tao, Yun Liang, Meng Ma, Dickson Adah, Wenting Ding, Lili Chen, Xiaofen Li, Linglin Dai, Songwe Fanuel, Siting Zhao, Wen Hu, Donghai Wu, Ziyuan Duan, Fang Zhou, Li Qin, Xiaoping Chen, Zhaoqing Yang

**Affiliations:** ^1^ Department of Medical Oncology, First Affiliated Hospital of Kunming Medical University, Kunming, Yunnan, China; ^2^ State Key Laboratory of Respiratory Disease, Center for Infection and Immunity, Guangzhou Institutes of Biomedicine and Health, Chinese Academy of Sciences (CAS), Guangzhou, Guangdong, China; ^3^ CAS-Lamvac (Guangzhou) Biomedical Technology Co., Ltd., Guangzhou, Guangdong, China; ^4^ Department of Hepatobiliary Surgery, First Affiliated Hospital of Kunming Medical University, Kunming, Yunnan, China; ^5^ The Ministry of Education Key Laboratory of Laboratory Medical Diagnostics, College of Laboratory Medicine, Chongqing Medical University, Chongqing, China; ^6^ Department of Applied Biosciences and Biotechnology, Faculty of Science and Technology, Midlands State University, Gweru, Zimbabwe; ^7^ Department of Pathogen Biology and Immunology, Kunming Medical University, Kunming, Yunnan, China

**Keywords:** *Plasmodium* immunotherapy, *Plasmodium chabaudi* ASS, gemcitabine, anticancer effect, synergism, mouse lung cancer model

## Abstract

**Objective:**

Our previous studies have demonstrated that *Plasmodium* immunotherapy (infection) has antitumor effects in mice. However, as a new form of immunotherapy, this therapy has a weakness: its specific killing effect on tumor cells is relatively weak. Therefore, we tested whether *Plasmodium* immunotherapy combined with gemcitabine (Gem), a representative chemotherapy drug, has synergistic antitumor effects.

**Methods:**

We designed subcutaneously and intravenously implanted murine Lewis lung cancer (LLC) models to test the antitumor effect of *Plasmodium chabaudi* ASS (Pc) infection in combination with Gem treatment and explored its underlying mechanisms.

**Results:**

We found that both Pc infection alone and Gem treatment alone significantly inhibited tumor growth in the subcutaneous model, and combination therapy was more effective than either monotherapy. Monotherapy only tended to prolong the survival of tumor-bearing mice, while the combination therapy significantly extended the survival of mice, indicating a significant synergistic effect of the combination. In the mechanistic experiments, we found that the combination therapy significantly upregulated E-cadherin and downregulated Snail protein expression levels, thus inhibiting epithelial-mesenchymal transition (EMT) of tumor cells, which may be due to the blockade of CXCR2/TGF-β-mediated PI3K/Akt/GSK-3β signaling pathway.

**Conclusion:**

The combination of Pc and Gem plays a synergistic role in inhibiting tumor growth and metastasis, and prolonging mice survival in murine lung cancer models. These effects are partially attributed to the inhibition of EMT of tumor cells, which is potentially due to the blockade of CXCR2/TGF-β-mediated PI3K/Akt/GSK-3β/Snail signaling pathway. The clinical transformation of *Plasmodium* immunotherapy combined with Gem for lung cancer is worthy of expectation.

## Introduction

Lung cancer is the most common cancer in the world, with the highest mortality and the second morbidity among all types of malignant tumors (1). Even though the traditional methods of surgery and chemoradiotherapy have improved, more than 40% of patients with non-small cell lung cancer (NSCLC) treated in early stage still have tumor recurrence and metastasis (2). In addition, about 65.33% of patients diagnosed with NSCLC are in the late stage (3), and the effect of conventional chemotherapy or radiotherapy is limited. Although some patients respond to targeted therapy, the development of drug resistance leads to tumor progression in a certain period (4). Since 2013, cancer immunotherapy represented by immune checkpoint inhibitors has been crowned as one of the breakthroughs of science and technology (5). However, some challenges remain to be addressed, including immune-related toxicities (6, 7), and primary/acquired resistance to therapy (8). There are no ideal and effective treatment models with little toxicity and the ability to inhibit tumor metastasis to improve the therapeutic effect of lung cancer patients so far.

Malaria is a disease caused by the infection of *Plasmodium* parasite, which is the most common parasitic infection in humans and animals. Studies have indicated that *Plasmodium* infection activates the immune system (9–11), which may help to repress tumor growth. Our previous studies have demonstrated that *Plasmodium* infection inhibits Lewis lung cancer (LLC) growth and metastasis by activating antitumor immune response (12), inhibiting angiogenesis (13, 14) and counteracting the tumor immunosuppressive microenvironment in mice (15). However, as a new form of immunotherapy, namely, *Plasmodium* immunotherapy, we still need to further enhance the specific killing effect of this therapy to increase its efficacy. In principle, chemotherapeutic drugs just have this effect, and gemcitabine (Gem) as a typical representative of these drugs is an effective and commonly used cytotoxic agent for the treatment of various tumors including NSCLC (16). Therefore, we designed murine NSCLC (LLC) models to examine the effects of *Plasmodium* infection in combination with Gem treatment on the inhibition of tumor growth and metastasis.

Tumor metastasis is a complex process that involves numerous factors and multiple steps, of which the epithelial-mesenchymal transition (EMT) is considered the initial and critical event in many types of carcinoma (17, 18). Chemokines, which are small chemoattractants in regulating cell positioning and cell recruitment into tissues, have been found to play an important role in cancer metastasis and EMT (19). Multiple signaling pathways participate in the progression of EMT, of which the chemokine-mediated PI3K/Akt/GSK−3β/Snail signaling is an essential process (20, 21). Therefore, in our current study, we tested whether *Plasmodium* infection in combination with Gem inhibited tumor metastasis and EMT, and whether these inhibitions were associated with the blocking of the above-mentioned pathways. Our results indicated that the combination of *Plasmodium* infection and Gem treatment significantly suppressed tumor metastasis and EMT, and these suppressions were potentially associated with the blockade of CXCR2/TGF-β-mediated PI3K/Akt/GSK-3β/Snail signaling pathway.

## Materials and methods

### Ethics statement

Our animal experiment facilities were approved by the Guangdong Provincial Department of Science and Technology. The animal experiments were approved by the Welfare Committee of the Center of Experimental Animals, Guangzhou Institutes of Biomedicine and Health (GIBH), Chinese Academy of Sciences (approval no. N2019014; Guangzhou, China), and strictly followed the standard guidelines for the care of animals. Animal suffering was minimized during the experiments.

### Animals, cells, and parasites

Six- to eight-week-old female C57BL/6 mice were purchased from Vital River Experiment Animal Limited Company (Beijing, China) and kept in a specific pathogen-free (SPF) animal facility of the GIBH. *Plasmodium yoelii* 17XNL (MRA-593, Py) and *Plasmodium chabaudi* ASS (MRA-429, Pc) strains were both donated by Malaria Research and Reference Reagent Resource Center (MR4). The murine LLC cells were obtained from ATCC and cultured with the medium containing Dulbecco’s modified Eagle’s medium (DMEM, Gibco), penicillin (80 U/ml), streptomycin (100 U/ml) and 10% fetal bovine serum (FBS, Gibco) in a humidified atmosphere of 5% CO_2_ at 37°C. Py and Pc were both intraperitoneally injected and propagated in C57BL/6 mice, respectively. Peripheral blood parasitemia was measured in thin blood smear by Giemsa staining (Sigma-Aldrich). Parasitemia (%) was calculated by counting the parasitized erythrocytes in at least 1000 erythrocytes.

### Subcutaneous tumor model and treatment

For the subcutaneous tumor model, 5×10^5^ LLC cells were subcutaneously (s.c.) inoculated into the right flank of C57BL/6 mice. Mice were randomly divided into four groups of 15 mice each according to body weight stratification: Control group (Con); Pc infection group (Pc); Gem treatment group (Gem); Pc infection combined with Gem treatment group (Pc+Gem). Mice were then intraperitoneally (i.p.) injected with 5×10^5^ Pc infected red blood cells on day 7 in the Pc group and Pc+Gem group. Mice were treated with Gem (50 mg/kg; 0.2 mL per time) i.p. for twice on day 6 and day 13 in the Gem group and Pc+Gem group. The tumor volume was recorded every two days after the tumor became palpable. The formula for calculating the volume is V = (ab^2^)/2, where “a” represents the long diameter, and “b” represents the short diameter of the tumor (12). Five mice randomly selected from each group were sacrificed on day 17 for calculating their body weight and tumor weight, and for analyzing their tumor tissues. The remaining mice were continually observed for their survival.

### Intravenous tumor model and treatment

For intravenous tumor model, 5×10^5^ LLC cells were intravenously (i.v.) injected into the C57BL/6 mice. Mice were randomly divided into four groups of 15 mice each: Con group, Pc group, Gem group, and Pc+Gem group. Each mouse of the Pc group and Pc+Gem group was i.p. injected with 5×10^5^ (Pc) parasitized erythrocytes on day 2. Each mouse in the Gem group and Pc+Gem group was (i.p.) given Gem (50 mg/kg, 0.2 ml per time) for twice on day 12 and 19. Five mice randomly selected from each group were sacrificed for counting individual metastatic nodules on the surface of lung under a microscope on day 32. The remaining mice continued to be observed for their survival.

### Protein extraction and Western blotting

Total protein was extracted from the tumor tissue using lysis buffer (RIPA, Beyotime) with protease inhibitor (Biotool) and phosphatase inhibitor cocktail (CST). Protein concentration was determined by BCA method. Total protein (50-100 μg) was separated by 10%-12% SDS-PAGE electrophoresis and then transferred to a PVDF membrane (Millipore). The membrane was blocked with 3% skim milk powder in tris-buffered saline with 0.1% tween 20 (TBST, Thermo Fisher Scientific, Inc) for 90 minutes at room temperature, and then it was incubated overnight with a solution containing the primary antibody. After washing with TBST for three times, the membrane was incubated at room temperature for 1 hour with horseradish peroxidase–conjugated secondary antibody diluted in TBST. Protein bands were observed by enhanced chemiluminescence (Pierce) and detected by BioImaging Systems (BIO-RAD ChemiDoc™ MP Imaging System, USA). GAPDH expression was used as a normalized protein.

### Reagents list used in the study

Antibodies: GAPDH (cat. no. ab9385; Abcam), E-cadherin (cat. no. BS1098; Bioworld Technology, Inc.), Snail (cat. no. 3879S; Cell Signaling Technology, Inc.), PI3K (cat. no. ab151549; Abcam), phosphorylated (p)PI3K p85 (Tyr458)/p55 (Tyr199) (cat. no. 4228S; Cell Signaling Technology, Inc.), Akt (cat. no. 9272S; Cell Signaling Technology, Inc.), pAkt (Ser473) (cat. no. 4060S; Cell Signaling Technology, Inc.), GSK 3β (cat. no. 12456S; Cell Signaling Technology, Inc.), pGSK 3β Ser9 (cat. no. 5558S; Cell Signaling Technology, Inc.), CXCR2 (cat. no. ab217314; Abcam) and TGF-β (cat. no. ab25121; Abcam).

### Statistical analysis

The survival was analyzed by Kaplan-Meier and compared by the Log-rank test. Data between groups were analyzed by Student’s t-test. *p* values less than 0.05, 0.01, and 0.001 were considered statistically significant and indicated by *, ** and ***, respectively, ns means “no significance” in each. All statistical analysis was performed using GraphPad Prism8.0 (GraphPad Software, Inc. https://www.graphpad.com/scientific-software/prism/).

## Results

### The combination of Pc with Gem significantly inhibited LLC subcutaneous tumor growth in mice

**Figure 1:**
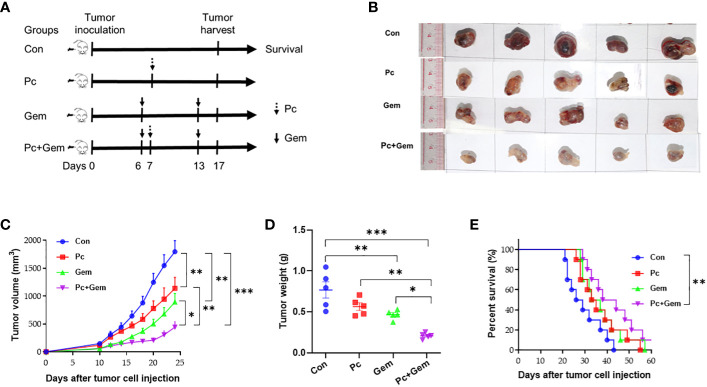
Pc+Gem combination treated lung cancer in the s.c. implanted murine LLC model. **(A)** Simplified experimental flow chart of s.c. implanted murine LLC model. **(B)** The image of tumor size on day 17 after tumor cells injection (n=5). **(C)** Tumor growth curves (n = 10 each group). **(D)** Tumor weight on day 17 after tumor cells injection (n = 5). The statistical differences were analyzed with an unpaired two-tailed Student’s t-test. **(E)** Kaplan-Meier survival curves (n = 10). Survival curves were analyzed by a log-rank test. *p* values less than 0.05, 0.01, and 0.001 were considered statistically significant and indicated by *, ** and ***, respectively.

To investigate the antitumor effect of the combination of Pc and Gem, subcutaneously implanted tumor-bearing mice were treated with Pc in combination with Gem (**Figure 1A**). The subcutaneous tumors of mice in Pc+Gem group had less blood supply, and looked like benign tumors (**Figure 1B**). Both Pc infection alone (*p* = 0.005) and Gem treatment alone (*p* = 0.002) significantly inhibited tumor growth in the subcutaneous model. Furthermore, tumor growth was significantly slower in the combination (Pc+Gem) group than in the control group(*p <* 0.001), and Pc alone (*p* = 0.006) or Gem alone (*p* = 0.03) group respectively (**Figure 1C**). The tumor weights and the ratios of tumor weight to the body weight in the combination group were also significantly lower than those in any other group (all *p* < 0.05) (**Figures 1D**, **S1**). The median survival time was 27.5 days, 34 days, 35 days and 41 days in the control group, Pc group, Gem group and Pc+Gem group, respectively (**Figure 1E**). Compared with the control, single treatment (Pc alone or Gem alone) only tended to prolong the life span of tumor-bearing mice without statistically significant differences in survival time between groups. Nevertheless, the life span of the combined treatment group was significantly longer than that of the control group (*p* = 0.007), suggesting a synergistic effect of the combination (**Figure 1E**).

### The combination of Pc with Gem significantly inhibited LLC metastasis in mice

**Figure 2:**
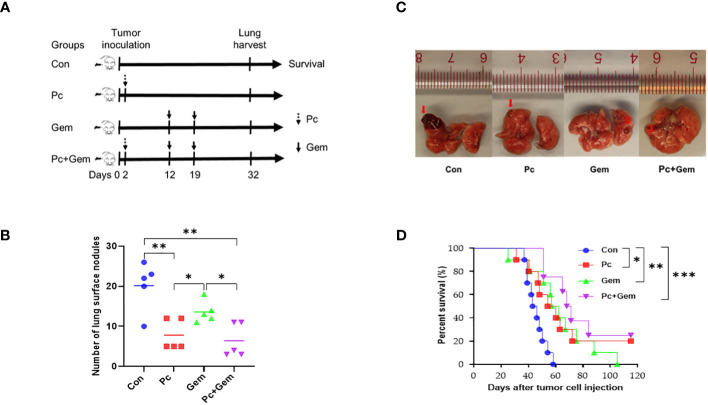
Pc+Gem treated lung cancer in the i.v. inoculated murine LLC model. **(A)** Simplified experimental flow chart of i.v. inoculated murine LLC model. **(B)** Number of tumor nodules in the lung tissues on day 32 after intravenous tumor inoculation (n = 5). The statistical differences were analyzed with an unpaired two-tailed Student’s t-test. **(C)** The representative image of tumor nodules in the lung tissues on day 32 after intravenous tumor inoculation. **(D)** Kaplan-Meier survival curve (n = 10). Survival curves were analyzed by a log-rank test. *p* values less than 0.05, 0.01, and 0.001 were considered statistically significant and indicated by *, ** and ***, respectively.

To determine the anti-metastatic effect of Pc+Gem, we established a mouse tumor model with intravenous injection of LLC cells (**Figure 2A**). The number of lung tumor nodules on day 32 in the Pc alone group or combination group was significantly less than that in the control group (*p* = 0.005 and *p* = 0.003, respectively) and Gem alone group (*p* = 0.02 and *p* = 0.02, respectively) (**Figure 2B**). These phenomena suggested that the role of Pc on the inhibition of metastasis was greater than that of Gem. In the control group, large transparent gelatinoid metastastic nodules were observed on the lung surface, some of which were accompanied by local bleeding. In the treatment groups, the metastases were smaller, and most of the metastases were only observed under magnifying glass after lung tissue dissection (**Figure 2C**). The median survival time was 44.5 days, 57 days, 59.5 days and 69.5 days in the control group, Pc group, Gem group and Pc+Gem group, respectively (**Figure 2D**). Accordingly, we also found that the mice in the Pc group, Gem group and Pc+Gem group survived significantly longer than the mice in the control group (*p* = 0.02, *p* = 0.006 and *p <* 0.001, respectively), the mice in the combination treatment group appeared to live longer than those in any monotherapy group, but the differences between the groups were not statistically significant (**Figure 2D**).

### The combination of Pc and Gem significantly inhibited the EMT of tumor cells

**Figure 3:**
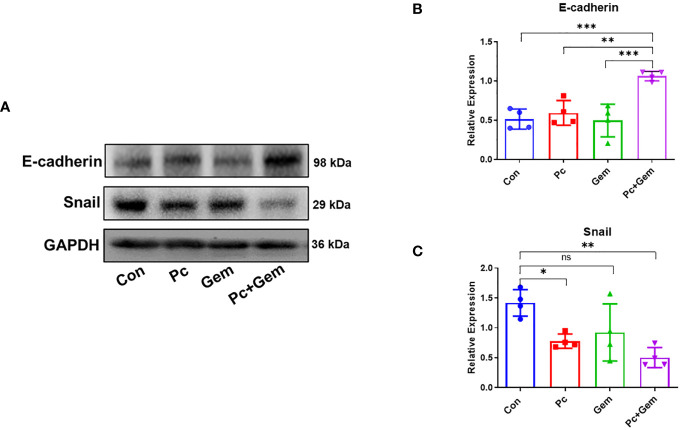
Pc+Gem significantly up-regulated E-cadherin and down-regulated Snail protein expression levels. The experimental scheme was shown in **Figure 1A**. **(A)** The results of Western blotting analysis of E-cadherin and Snail in LLC subcutaneous tumors on day 17 after tumor cells injection. **(B)** The relative expression of E-cadherin/GAPDH (n = 4). **(C)** The relative expression of Snail/GAPDH (n = 4). The statistical differences were analyzed with an unpaired two-tailed Student’s t-test. *p* values less than 0.05, 0.01, and 0.001 were considered statistically significant and indicated by *, ** and ***, respectively. *p* values more than 0.05 were considered statistically non-significantly and indicated by ns.

Tumor cells obtain an invasive phenotype for metastatic progression through EMT, which allows them to dissociate from the primary tumor into the blood circulation (22). The loss of E-cadherin expression has been considered a landmark for EMT (23). Our experimental results showed that there were no significant differences in the protein expression level of E-cadherin between each monotherapy group and the control group, but the level of this molecule in the combination treatment group was significantly higher than that in the control group or that in Pc group and Gem group (*p <* 0.001, *p* = 0.002 and *p <* 0.001, respectively) (**Figures 3A, B**), suggesting a significantly synergistic effect of the combined treatment. The Snail superfamily of Zinc-finger transcription factors is a crucial transcription inhibitor for EMT, which can directly lead to inhibition of E-cadherin (24). Our results indicated that the expression level of Snail protein in the Pc group and Pc+Gem group was significantly lower than that in the control group (*p* = 0.02 and *p* = 0.002, respectively). Although there were no significant differences between the treatment groups, the level of this molecule in Gem group was not statistically significant than that in the control group, which suggested that combination treatment group has a synergistic effect on the inhibition of Snail protein expression and the role of Pc on the inhibition of Snail protein expression was greater than that of Gem (**Figures 3A, C**).

### The combination of Pc and Gem significantly suppressed the activation of PI3K/Akt/GSK-3β signaling pathway

**Figure 4:**
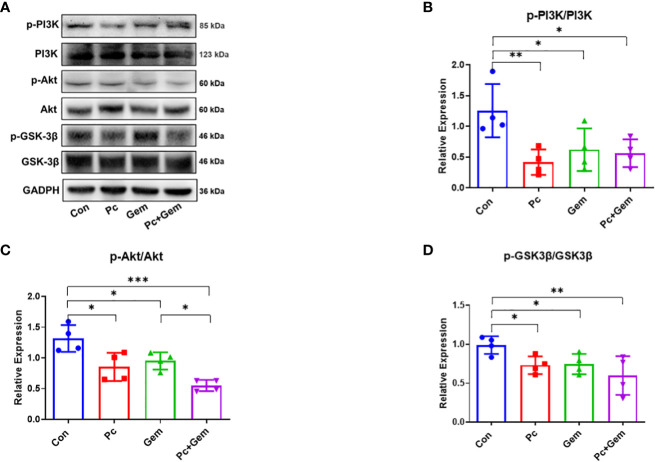
Pc+Gem significantly inhibited the activation of the PI3K/Akt/GSK-3β signaling pathway. The experimental scheme was shown in **Figure 1A**. **(A)** The results of Western blotting analysis of PI3K/Akt/GSK-3β in LLC subcutaneous tumors on day 17 after tumor cells injection. **(B)** The relative expression of p-PI3K/PI3K (n = 4). **(C)** The relative expression of p-Akt/Akt (n = 4). **(D)** The relative expression of p-GSK-3β/GSK-3β (n = 4). The statistical differences were analyzed with an unpaired two-tailed Student’s t-test. *p* values less than 0.05, 0.01, and 0.001 were considered statistically significant and indicated by *, ** and ***, respectively.

Some studies have indicated that the PI3K/Akt/GSK-3β signaling pathway plays an important role by affecting the activity of the transcription factor, Snail that is related to EMT in many types of tumors (20, 21). Therefore, we examined PI3K/Akt/GSK-3β protein expression levels in the tumors of these mice. The results of Western blotting analysis indicated that the phosphorylation level of PI3K in any treatment group (Pc, Gem, or Pc+Gem) was lower than that in the control group (*p* = 0.007, *p* = 0.04 and *p* = 0.02, respectively), but there were no significant differences between the treatment groups, suggesting that the combination of the two treatments had neither synergistic nor antagonistic effect on this molecule (**Figures 4A, B**). Similarly, the phosphorylation level of Akt in any treatment group (Pc, Gem, or Pc+Gem) was also lower than that in the control group (*p* = 0.02, *p* = 0.03 and *p <* 0.001, respectively). Furthermore, the phosphorylation level of Akt in Pc+Gem group was significantly lower than the Gem group (*p* = 0.02). At the same time, the expression level of phosphorylated Akt in the Pc+Gem group also appeared to be lower than the Pc group, but the difference between the two groups was not statistically significant. These results suggested that the combination of the two treatments had a synergistic effect on the expression of this molecule (**Figures 4A, C**). The phosphorylation level of GSK-3β in any of the treatment groups (Pc, Gem, or Pc+Gem) was lower than that in the control group (*p* = 0.02, *p* = 0.03 and *p =* 0.005, respectively), but there were no significant differences between the treatment groups, suggesting that the combination of the two treatments did not have a synergistic effect on the expression of this molecule, neither did it have an antagonistic effect (**Figures 4A, D**).

### The combination of Pc and Gem significantly downregulated the expression of CXCR2/TGF-β

**Figure 5:**
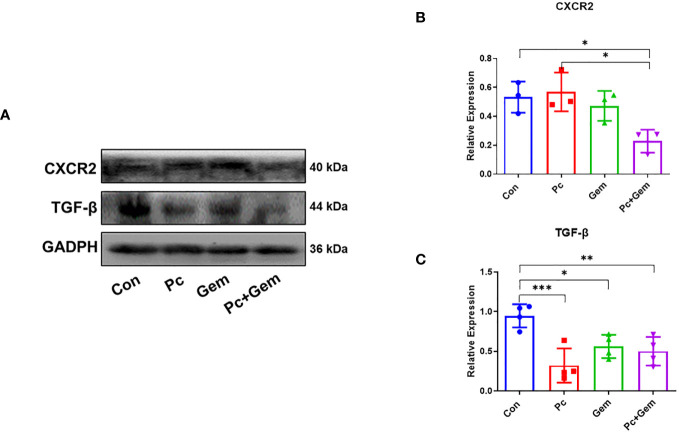
Pc+Gem significantly down-regulated the protein expression levels of CXCR2/TGF-β. The experimental scheme was shown in **Figure 1A**. **(A)** The results of Western blotting analysis of CXCR2/TGF-β in LLC subcutaneous tumors on day 17 after tumor cells injection. **(B)** The relative expression of CXCR2/GAPDH (n = 3). **(C)** The relative expression of TGF-β/GAPDH (n = 4). The statistical differences were analyzed with an unpaired two-tailed Student’s t-test. *p* values less than 0.05, 0.01, and 0.001 were considered statistically significant and indicated by *, ** and ***, respectively.

CXC-chemokine receptor 2 (CXCR2) has been demonstrated to have the ability to activate PI3K/Akt/GSK-3β/Snail signaling pathways and promote cell migration (25, 26). Therefore, we examined the expression of these molecules in tumor tissues of mice. The Western blotting results showed that there were no significant differences in CXCR2 protein expression level between any single treatment (Pc or Gem) group and the control group, but the level of this molecule in the Pc+Gem group was significantly lower than that in the control group (*p* = 0.03). In addition, the expression level of this molecule in the Pc+Gem group was also significantly lower than that in the Pc group (*p* = 0.02). These phenomena suggested that the combination of the two treatments had a synergistic effect on this molecule (**Figures 5A, B**). We further examined the expression level of TGF-β which has also been shown to promote EMT through PI3K/Akt/GSK-3β/Snail signaling (27). Our results indicated that the expression level of TGF-β protein in any treatment (Pc, Gem or Pc+Gem) group was significantly lower than that in the control group (*p <* 0.001, *p* = 0.03 and *p =* 0.003, respectively), but there were no significant differences between the treatment groups, suggesting that the combined treatment had no synergistic effect, neither did it show any antagonistic effect on the expression of this molecule (**Figures 5A, C**).

## Discussion

Our current study indicates that the combination of *Plasmodium* (Pc) infection with Gem treatment has a synergistic effect on the inhibition of tumor growth and prolongation of survival in a subcutaneously inoculated mouse lung cancer model (**Figure 1**) and a synergistic effect on the inhibition of tumor metastasis and prolongation of survival in an intravenously inoculated mouse tumor model (**Figure 2**). Pc infection may contribute more than the Gem treatment in the inhibition of tumor metastasis (**Figure 2B**). These results are consistent with those of our previous studies that *Plasmodium yoelii* 17XNL (Py) infection significantly reduces tumor metastasis and recurrence after the original tumor is removed through operation in a murine liver cancer model (21), and that the combination of Py infection with DNA cancer vaccine treatment has a synergistic antitumor effect in a murine lung cancer model (12).

Studies have shown that tumor metastasis is highly related to EMT of tumor cells and the apparent characteristics of EMT are a downregulation of E-cadherin and an upregulation of Snail in tumor cells (17–19). Our current results have demonstrated that Pc infection in combination with Gem treatment significantly up-regulates the protein expression level of E-cadherin and down-regulates the protein expression level of Snail in tumor tissues (**Figure 3**), which indicates a significant inhibition of EMT. Even though Gem alone may induce EMT in patient-derived pancreatic ductal adenocarcinoma xenografts (28), we did not observe a similar effect represented by the expression levels of E-cadherin and Snail in our current murine lung cancer model (**Figure 3**).

**Figure 6:**
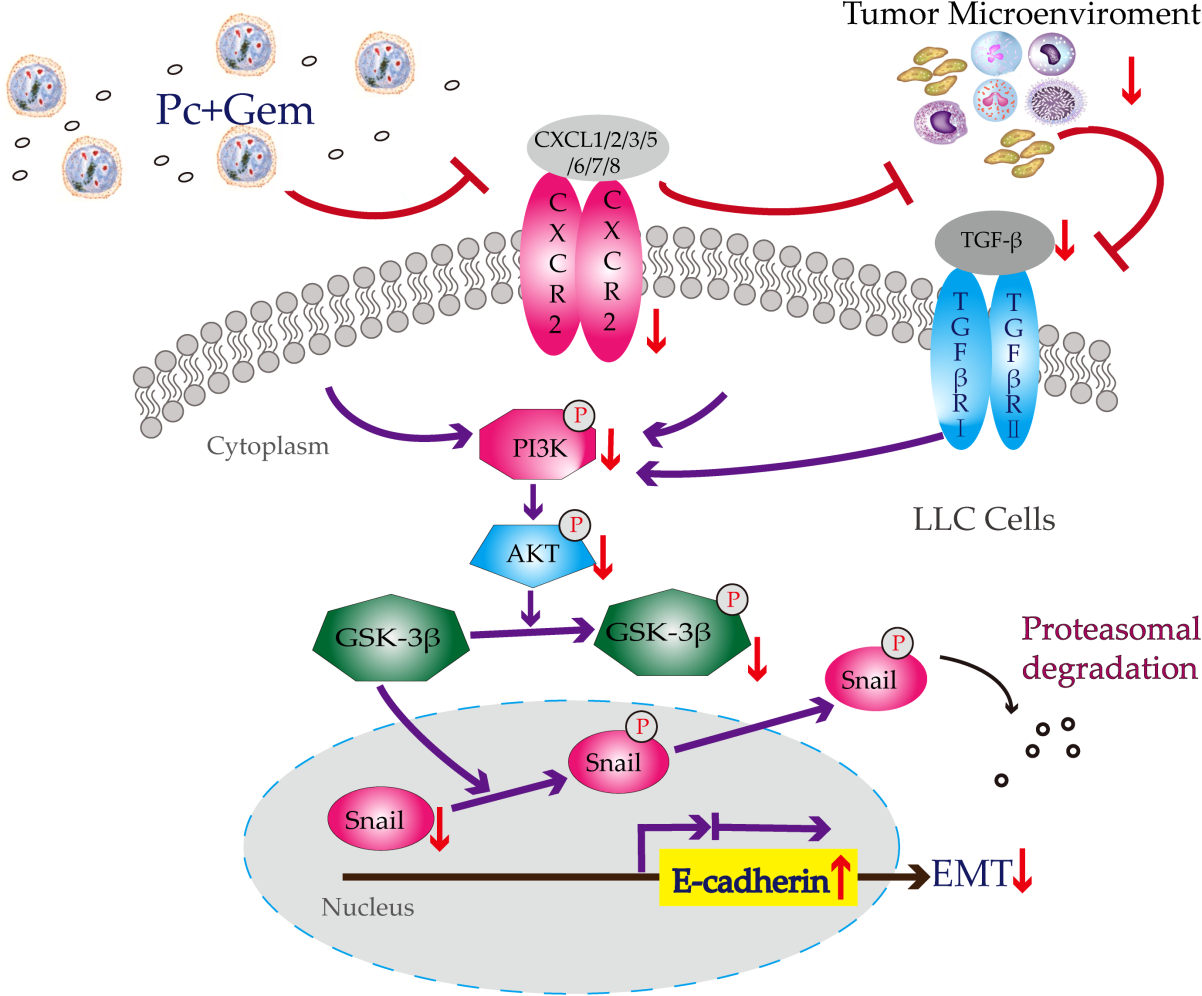
Potential mechanism of action of Pc+Gem in the inhibition of tumor cell EMT in murine Lewis lung cancer models. Pc+Gem induces: 1) down-regulation of CXCR2/TGF-β; 2) down-regulation of p-PI3K, but not PI3K, suggesting the inhibition of the phosphorylase of PI3K; 3) downregulation of p-Akt, but not Akt, suggesting the inhibition of the phosphorylase of Akt; 4) down-regulation of p-GSK-3β, but not GSK-3β, suggesting the inhibition of the phosphorylase of GSK-3β; 5) down-regulation of Snail; 6) up-regulation of E-cadherin. The down-regulation of Snail and up-regulation of E-cadherin are the main characteristics of suppression of the EMT in murine LLC models.

Our previous study has suggested that Py infection inhibits PI3K/Akt/GSK-3β/Snail signaling pathway and therefore inhibits EMT in a murine liver cancer model (21). In our current study, we tested whether the combination of Pc and Gem or Pc infection alone or Gem treatment alone inhibited this pathway in a murine lung cancer model. The results show that the combination or Pc infection alone or Gem treatment alone indeed inhibits this pathway (**Figure 4**). Then we asked whether CCR10 mediated this pathway as demonstrated in our previous study of a liver cancer model infected with Py (21). The result suggested that the CCR10 protein level in lung cancer tissue was not affected by Pc infection, or Gem treatment or the combination of both (**Figure S2**). Then we tested the protein expression levels of CXCR2 and TGF-β, because some studies have indicated that CXCR2 can also mediate PI3K/Akt/GSK-3β/Snail signaling pathway (25, 26), and many researches have revealed that CXCR2 is also significant in the recruitment of different cells (tumor-associated macrophages, tumor-associated neutrophils, myeloid-derived suppressor cells, regulatory T cells) which constitute the tumor microenvironment (29–32). These components can cause EMT by regulating TGF-β secretion which can be involved in PI3K/Akt/GSK-3β pathway (27). The results show that the combination of Pc and Gem treatment significantly downregulates the protein level of CXCR2 compared with the control group, but Pc alone or Gem alone does not affect its level (**Figures 5A, B**). Finally, our current results show that Pc infection alone or Gem treatment alone or the combination of both significantly inhibits the protein expression level of TGF-β (**Figures 5A, C**). Based on the results mentioned above, we summarize the possible mechanism of action of Pc infection in combination with Gem treatment on EMT of tumor cells in murine lung cancer models as shown in **Figure 6**.

Studies have suggested that high EMT score of tumor cells promotes immune evasion, while low EMT score promotes antitumor immune response, and vice versa, an effective antitumor immune response can inhibit EMT and kill tumor cells at the same time (33–35). Other studies have pointed out that STAT3 signaling plays an important role in the occurrence and development of EMT, and the interaction between STAT3 and TGF-β not only induces EMT, but also serves as the central regulatory signal molecules for the formation of immunosuppressive network in the tumor microenvironment (36–39). Our previous study in mouse tumor models shows that *Plasmodium* infection significantly downregulates the phosphorylated level of STAT3 (pSTAT3) within MDSCs in tumor tissues and the expression level of TGF-β in tumor tissues, thus releasing the tumor immunosuppressive microenvironment (15). Combined with the findings of current study, namely, *Plasmodium* infection blocks TGF-β-induced EMT, we believe that the activation of the immune system of the tumor-bearing host, the relief of the immunosuppressive state, and the inhibition of EMT by *Plasmodium* infection, are highly unified because they share certain signaling pathways and key targets. By inhibiting these targets and pathways, *Plasmodium* infection can promote the body’s anti-tumor immune response, inhibit tumor growth and metastasis, and thus prolong the life of tumor-bearing hosts.

However, there have been many conflicting results and controversies regarding whether *Plasmodium* infection suppresses or activates the immune system (40–42). Urban BC et al. proposed a hypothesis to reconcile these conflicting results, where they suggested that high density of parasitemia or high concentration of malaria pigment (hemozoin) inhibited dendritic cells (DC) and downstream T and B cells, while low density of parasitemia or low concentration of hemozoin activated DC and its downstream immune cells (43). Nevertheless, what we observed in *Plasmodium*-infected tumor-bearing mice is inconsistent with this hypothesis. First, we compared the parasitemia induced by Py and Pc. In both tumor-free mice and lung cancer-bearing mice, the parasite density (red blood cell infection rate) induced by Py was significantly higher than that induced by Pc, and the duration of high parasitemia was significantly longer in Py than in Pc (**Figure S3**). Then, we compared the inhibitory effect of Py and Pc on lung cancer in mice and found that the effect of Py was significantly better than that of Pc (**Figure S4**). Finally, we observed a positive correlation of parasite density with spleen size (represents the degree of immune response to the infection) and a negative correlation of parasite density with tumor size (**Figure S5**), suggesting that *Plasmodium* infection could induce antitumor immune response in a parasitemia-dependent manner. In fact, one of the best ways to test whether *Plasmodium* infection activates or suppresses the immune system is to observe it in a tumor-bearing host, because the immune system of the tumor-bearing host is inhibited by tumor cells, if it suppresses the immune system, it would promote tumor growth, if it activates the immune system, it would inhibit tumor growth. In brief, what we observed in the tumor-bearing hosts is that *Plasmodium* infection generally activates rather than suppresses the immune system, when the immune system has already been suppressed by the tumor cells. This has also been preliminarily demonstrated in our clinical trial of *Plasmodium* immunotherapy for advanced solid tumors. We used a relatively benign form of human parasite, namely, *Plasmodium vivax* (Pv), in clinical trials, and the parasitemia level of natural Pv infection in humans is fairly low compared to Py or Pc in mice (44). Furthermore, to ensure the safety of clinical trials, we used artesunate to control the parasitemia to very low levels (0.1% or less), and the low parasitemia has been shown to activate the immune system of patients with advanced cancer (45) and therefore has effect on the treatment of clinical tumors (unpublished data). Our clinical data preliminarily suggest that high parasitemia not only increases toxicity, but is also positively associated with poor prognosis in cancer patients (unpublished data), so we must strictly control the infection rate of the parasite. It is very important that the parasite that we have selected is a strain of *Plasmodium* vivax that is sensitive to all current antimalarial drugs, especially artesunate, and a single low dose of artesunate administered intravenously in a very short period of time can control the parasite density to less than 0.1%, so the side effects of *Plasmodium* immunotherapy are limited and manageable.

It is worthy of note that in our previous study, Py infection inhibits the CCR10-mediated PI3K/Akt/GSK-3β/Snail signaling pathway in a murine liver cancer model, but in our current study, Pc infection inhibits the CXCR2/TGF-β-mediated PI3K/Akt/GSK-3β/Snail signaling pathway in murine lung cancer models. There are two possible reasons for these differences. (1) Different murine cancer models: liver cancer versus lung cancer; (2) different murine *Plasmodium* parasites: Py versus Pc. This merits further study.

A series of previous studies conducted by us have shown that *Plasmodium* infection plays anticancer roles through multiple targets and multiple pathways, for example, it activates the innate and adaptive antitumor immune responses (12, 46); systematically removes the tumor immunosuppressive microenvironment through *Plasmodium*-associated exosomes that inhibit tumor cell secretion of cytokines and chemokines which have the ability to recruit the precursors of immune suppressor cells including myeloid-derived suppressor cells (MDSC) and regulatory T cells (Treg) into tumor tissue, thereby significantly down-regulating the number of these cells and inhibiting their function through undefined mechanisms (15); inhibits tumor angiogenesis through micro-RNA (miRNA) 16/322/497/17 within exosomes and long noncoding RNA (lncRNA F66), both of which target VEGF/VEGFR2 pathway via different mechanisms of action or through changing the functional phenotype of tumor-associated macrophages (TAM) via engulfing hemozoin that blocks IGF-1/MMP9 signal pathways (13, 14, 47), and inhibits EMT of tumor cells (21). By comparing *Plasmodium* immunotherapy with a single-target anticancer immunotherapy known as immune checkpoint blockade, we propose the notion that *Plasmodium* immunotherapy is an ecological therapy that systemically targets cancer as an ecological disease (45). However, *Plasmodium* immunotherapy also has an obvious shortage, that is, the specific killing of tumor cells is relatively weak, therefore, needs to be combined with other therapies to further improve its efficacy. Our current study has preliminarily confirmed that *Plasmodium* (Pc) infection combined with Gem treatment has a synergistic effect on the inhibition of EMT and tumor metastasis, and the prolongation of survival in lung cancer-bearing mice, without significantly enhancing their toxicity (**Figure S6**). Since Gem treatment induces immunogenic death of cancer cells (48) and inhibits MDSC, Treg, and TGF-β (49), Pc infection in combination with Gem may also have synergistic effects on immune killing of tumor cells. This merits further study. Nevertheless, since both *Plasmodium* infection and Gem treatment exert anticancer effects through multiple targets and multiple pathways, their synergistic effects on the overall anticancer effect, that is, inhibiting tumor growth and metastasis and prolonging the life span of tumor-bearing mice, do not mean that they must have synergistic effects on every signaling pathway and every target. Importantly, antagonism between them was not observed throughout the study.

There are some shortcomings in this study: we were unable to use some specific gene knockout mice for experiments. For example, IFN-γ, an inflammatory cytokine, is important for both anti-tumor immunity (50, 51) and anti-*Plasmodium* immunity, especially for its antagonistic effect on anti-inflammatory cytokine TGF-β (52). However, IFN-γ knockout mice would die when infected with *Plasmodium* parasites (53). Therefore, we are currently designing single-cell transcriptome studies to look for important target genes for *Plasmodium* immunotherapy.

In summary, we report for the first time the antitumor results of Pc alone and the synergistic effects of Pc combined with Gem in murine lung cancer models in our present study. These results indicate that Pc infection in combination with Gem treatment significantly inhibits tumor growth and metastasis, and prolongs the survival of lung cancer-bearing mice partially through inhibiting EMT of tumor cells that is possibly related to the blockade of CXCR2/TGF-β-mediated PI3K/Akt/GSK-3β/Snail signaling pathway. Based on the results of our series of preclinical studies in murine tumor models and our epidemiological data analysis showing a significant negative correlation between global malaria incidence and tumor mortality (54), clinical trials of *Plasmodium* immunotherapy for advanced cancer have been approved and are ongoing in China (NCT02786589, NCT03474822 and NCT04165590). Our current study provides a candidate for future clinical trials of *Plasmodium* immunotherapy in combination with other therapies for the treatment of cancer.

## Data availability statement

The original contributions presented in the study are included in the article/**Supplementary Material**. Further inquiries can be directed to the corresponding authors.

## Ethics statement

The animal study was approved by Guangzhou Institutes of Biomedicine and Health. The study was conducted in accordance with the local legislation and institutional requirements.

## Author contributions

XC and ZT conceived, planned and carried out this work. YL, MM, DA, LC, WD, XL, LD, SF, and SZ helped in the animal experiments. WH, DW, ZD, and FZ performed consultation and analyzed the results. LQ, XPC, and ZY supervised the research project. XPC and ZY revised the manuscript. All authors discussed the results and contributed to this work. All authors read and approved the final manuscript.
