# Supplementary material for: Plasmodium immunotherapy combined with gemcitabine has a synergistic inhibitory effect on tumor growth and metastasis in murine Lewis lung cancer models
Source: Front Oncol. 2023 Oct 17;13:1181176. doi: 10.3389/fonc.2023.1181176 (PMC10618005; doi:10.3389/fonc.2023.1181176)
Supplement: Supplementary file 1 [file DataSheet_1.docx]

**Supplemental Figures**

**
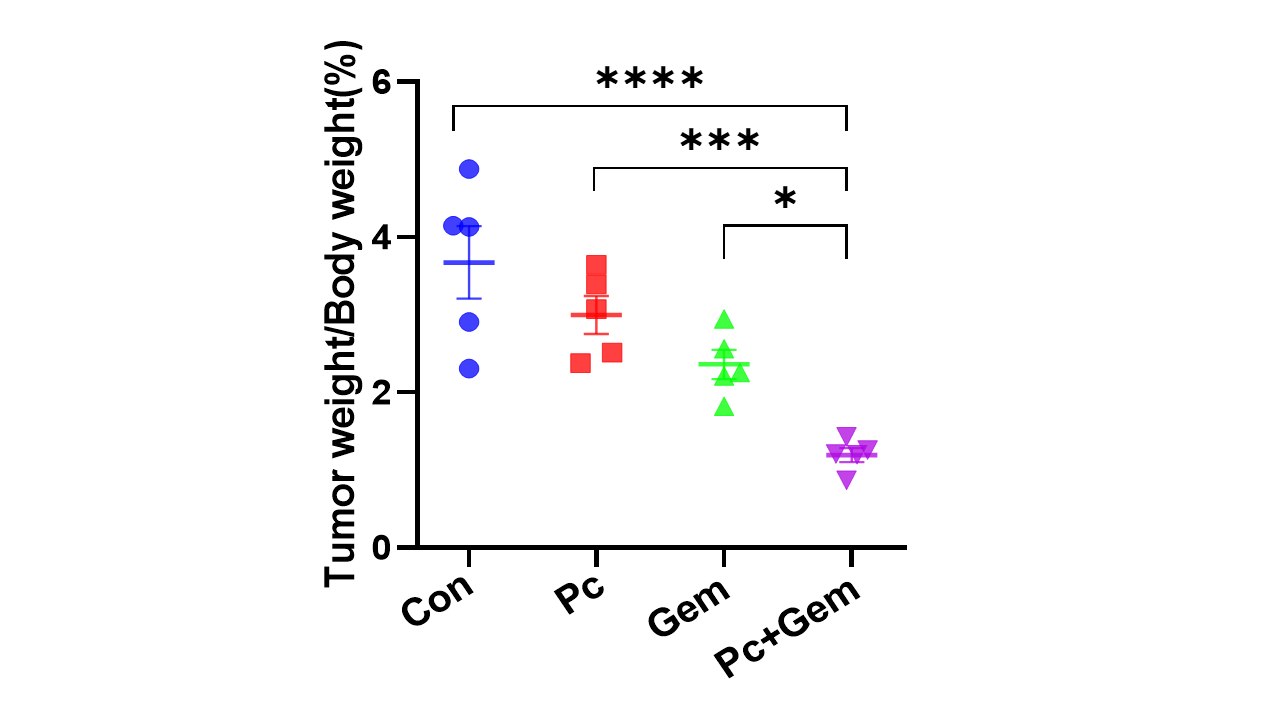
**

**Supplemental Figure S1**

Comparisons of the tumor weight and body weight ratio between groups in s.c. implanted murine LLC model. The percentage of tumor weight to body weight in Pc+Gem group was significantly lower than that in the other three groups, suggesting that the inhibition of tumor growth by Pc+Gem was not affected by weight loss in mice.

**
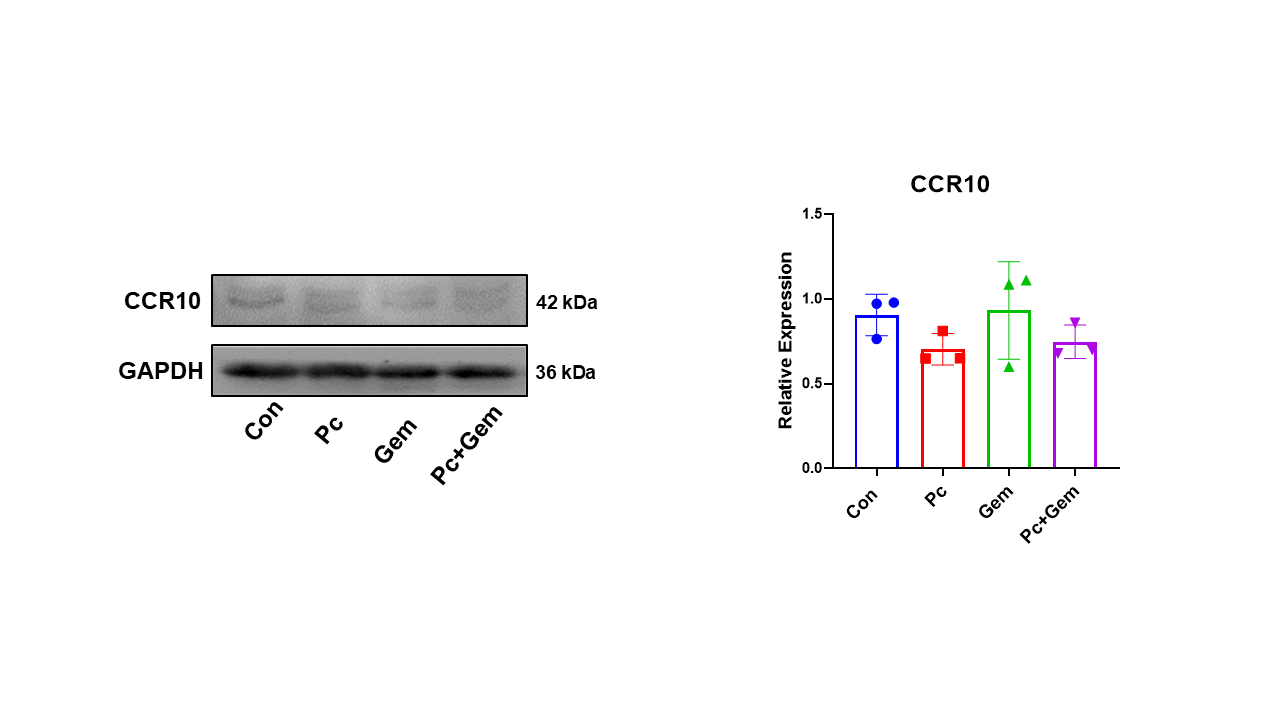
**

**Supplemental Figure S2**

The CCR10 protein level in LLC tissue was not significantly affected by Pc, Gem or Pc+Gem. The experimental scheme was shown in Figure 1A. (A) The results of Western blotting analysis of CCR10 in LLC primary tumors on day 17 after tumor cells injection. (B) The relative expression of CCR10/GAPDH (n = 3). The statistical differences were analyzed with an unpaired two-tailed Student’s t-test.


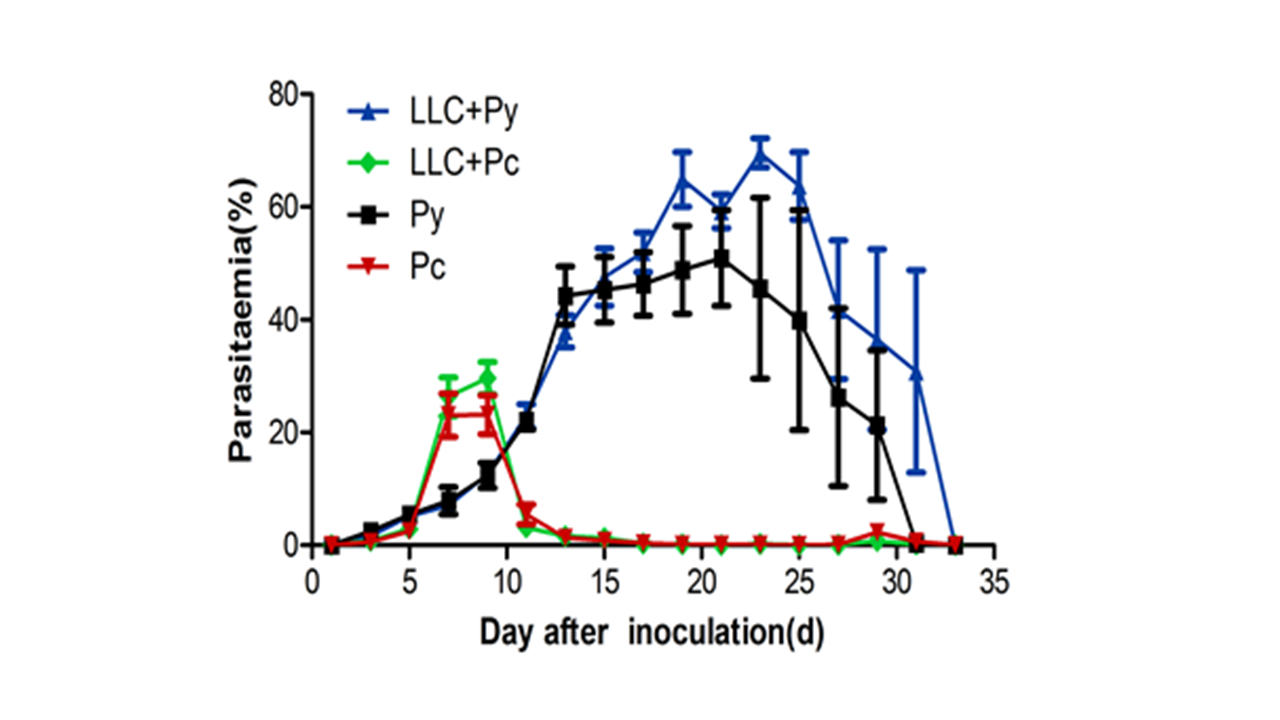


**Supplemental Figure S3**

Comparisons of parasitemia induced by Py and Pc. LLC+Py: Py-induced parasitemia in (LLC) lung cancer-bearing (C57BL/6) mice; LLC+Pc: Pc-induced parasitemia in lung cancer-bearing mice; Py: Py-induced parasitemia in naive (non-tumor) mice; Pc: Pc-induced parasitemia in naive mice.


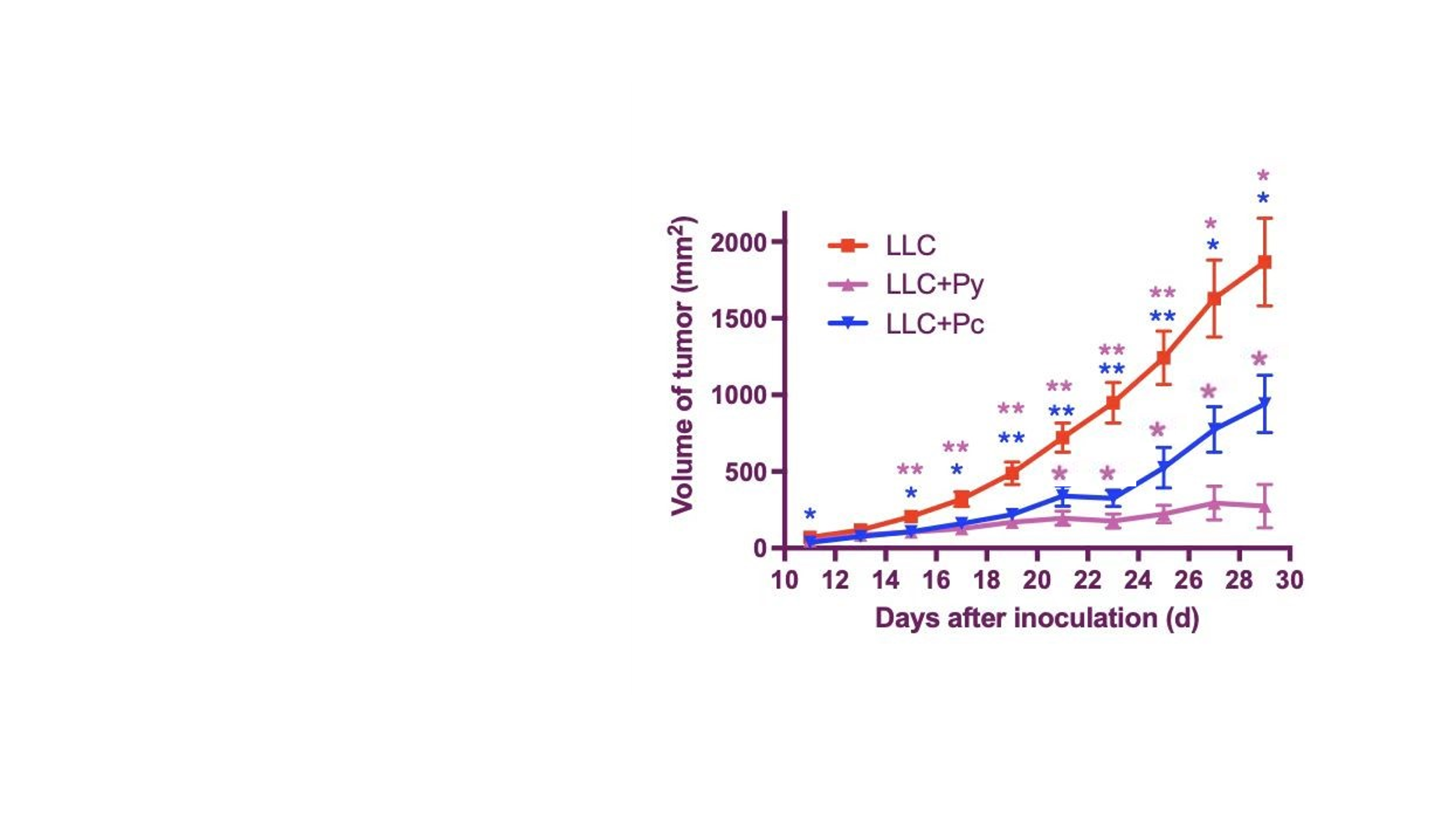


**Supplemental Figure S4**

Comparisons of inhibitory effect on tumor growth between Py and Pc infections in (C57BL/6) mice. LLC: (LLC) Tumor growth curve in (C57BL/6) mice without Plasmodium infection; LLC+Py: tumor growth curve in Py-infected mice; LLC+Pc: tumor growth curve in Pc-infected mice. * *p* < 0.05; ** *p* < 0.01.

**
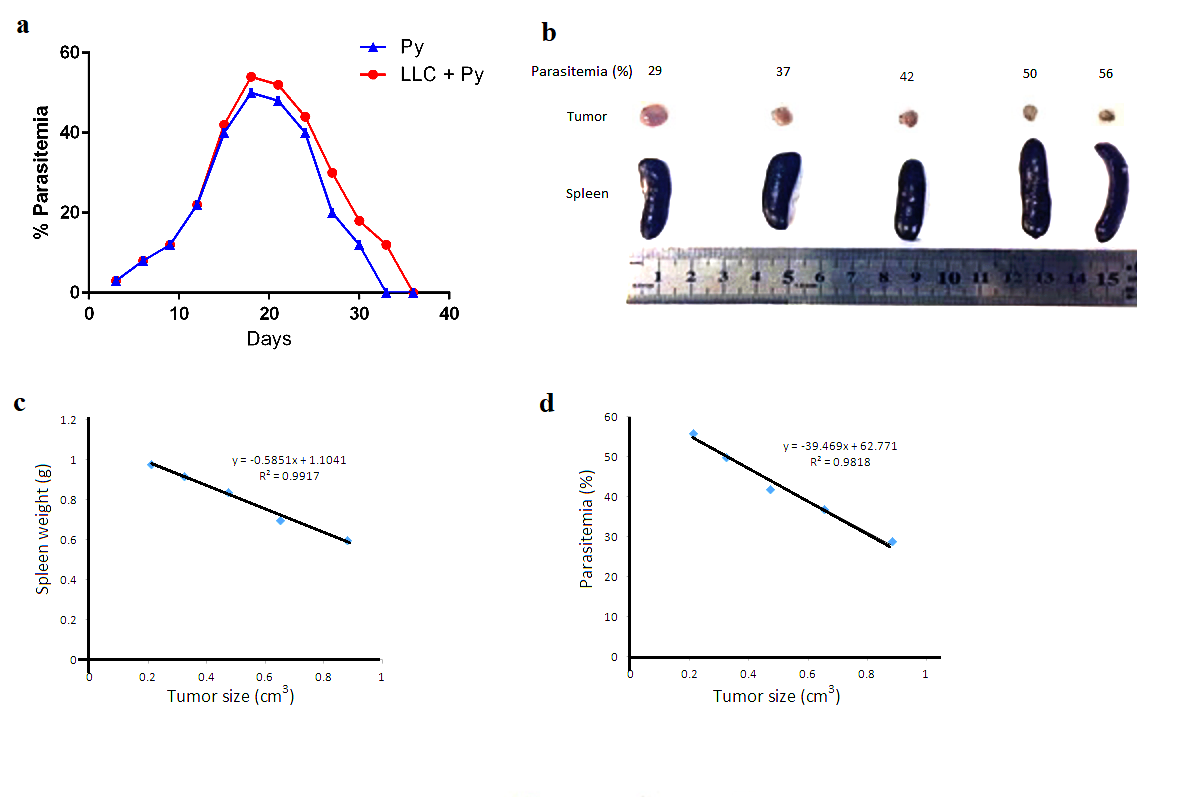
**

**Supplemental Figure S5**

Correlations of parasitemia level with spleen size and tumor size in Py-infected (LLC) lung cancer -bearing (C57BL/6) mice. (a) Comparison of parasitemia between Py-infected naïve (no tumor) mice (Py group) and tumor-bearing mice (LLC+Py group). (b, c & d) Mice with lower parasitemia had smaller spleen size (represents the degree of systemic immune responses) and bigger tumor size, while mice with higher parasitemia had bigger spleen size and smaller tumor size. The graph of tumor size vs parasitemia showed that the higher the parasitemia, the lower the tumor size. These results suggested that Py infection could induce anti-tumor immune response in a parasitemia-dependent manner.


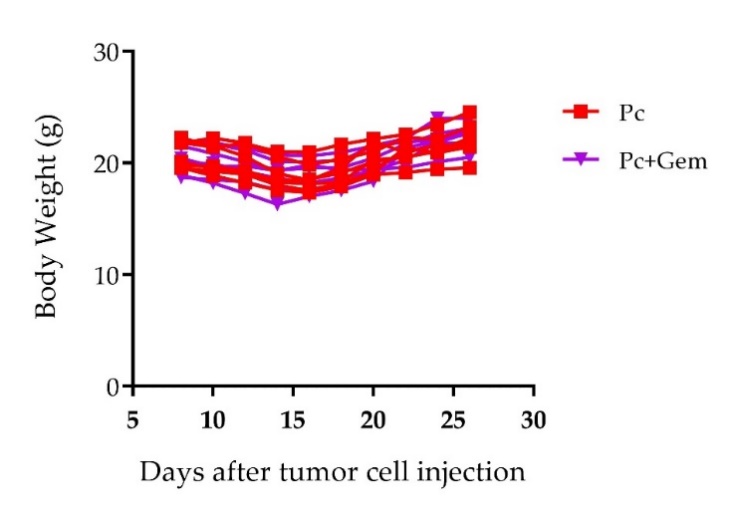


**Supplemental Figure S6**

The dynamic changes of body weight of the tumor-bearing mice (subcutaneous inoculation model). There were no significant differences between the two groups. Note: Pc in this figure represents the Pc group in the main text; Pc+Gem represents the Pc+Gem group in the main text.
